# Advances in the Knowledge of the Reproductive Processes of the Critically Endangered *Pinna nobilis* Linnaeus, 1758

**DOI:** 10.3390/biology15110847

**Published:** 2026-05-28

**Authors:** Emilio Cortés Melendreras, Pilar Martínez-Martínez, Juan Vera Inglés, Miguel Ángel Sánchez, Antonio Crespo Montalt, Yolanda Fernández-Torquemada, Ezequiel Martínez Ortega, Francisca Giménez Casalduero

**Affiliations:** 1 Aquarium of the University of Murcia, University of Murcia, 30002 Murcia, Spain; ecortes@um.es (E.C.M.); juan.vera1@um.es (J.V.I.); msanchez@um.es (M.Á.S.); antonioluis.crespo@um.es (A.C.M.); ezequiel@um.es (E.M.O.); 2 Department of Marine Sciences and Applied Biology, University of Alicante, 03690 San Vicente del Raspeig, Spain; yolanda.fernandez@ua.es (Y.F.-T.); francisca.gimenez@ua.es (F.G.C.); 3 Marine Research Centre of Santa Pola, University of Alicante, 03130 Santa Pola, Spain

**Keywords:** fan mussel, ex situ maintenance, reproduction, gonadal maturation, gamete release, fertilization

## Abstract

As part of conservation efforts for *Pinna nobilis*, a critically endangered bivalve endemic to the Mediterranean Sea, laboratory programs have been developed to maintain and breed specimens. However, progress in the ex situ conservation of the species remains limited and challenging. This study aims to advance the knowledge required to establish effective reproductive protocols for *P. nobilis*, specifically focusing on the population in the Mar Menor lagoon, one of the last two surviving populations along the Spanish coast. The first phase of this study involved characterizing the reproductive events in the lagoon. Subsequently, two ex situ reproduction experiments were conducted under conditions designed to replicate the lagoon’s natural environment. Three reproductive events were detected in the lagoon between 2019 and 2022, and five successful spawning events occurred across the two ex situ experiments. The conditions for maintenance, maturation, and induction of the individuals are described. In all cases, the percentage of fertilized oocytes released was remarkably high, suggesting internal fertilization, but not self-fertilization, within the pallial cavity. Additionally, ex situ individuals exhibited simultaneous hermaphroditism, with synchronous maturation and alternating release of gametes, effectively preventing self-fertilization. These findings represent a significant step forward in understanding the reproductive biology of *P. nobilis* and contribute to efforts aimed at ensuring the species’ long-term survival.

## Simple Summary

The fan mussel (*Pinna nobilis*) is a critically endangered bivalve species found in the Mediterranean Sea. Urgent action is needed to ensure its survival. This study focused on the remaining population in Spain’s Mar Menor lagoon. First, the species’ natural reproductive period in the lagoon was identified. Then, this information was used to carry out breeding experiments in a laboratory setting that replicated these conditions. Several successful spawning events were achieved. The study revealed that individuals mature gametes simultaneously but release male and female gametes at different times, which may help avoid self-fertilization. Very high fertilization rates were observed, suggesting that fertilization occurs internally, which improves our understanding of how this species reproduces. These results provide valuable insights for future conservation and breeding programs aimed at safeguarding the species.

## 1. Introduction

**Figure 1:**
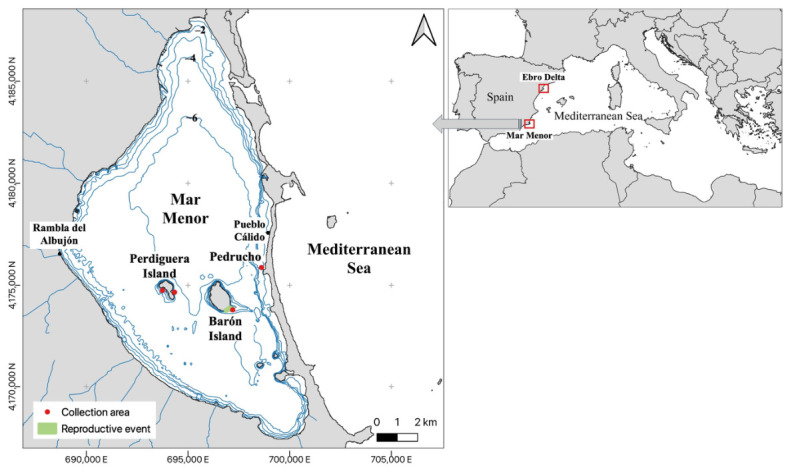
Right: Location of the two last surviving populations of *P. nobilis* in Spain (western Mediterranean Sea), Ebro Delta and Mar Menor (red boxes). Left: Study area, Mar Menor coastal lagoon (SE Spain). Broodstock extraction localities are shown in red. Locations of in situ reproductive events in green.

*Pinna nobilis* Linnaeus, 1758, is a Mediterranean bivalve affected by a mass mortality event (MME) since 2016 [1,2]. This MME was caused mainly by the protozoan *Haplosporidium pinnae* [3,4], although other pathogens could have played an important role in the disease event, such as bacteria of the genera *Vibrio* and *Mycobacterium* [5] or a Picornavirus that weakens its immune system [6,7]. Mortality rates close to 100% of individuals prompted a new status for the species: “Critically Endangered” [8,9]. Surviving populations have been relegated to areas where salinity levels are outside the survival range of *H. pinnae* [2]. The only two surviving populations of *P. nobilis* on the Spanish coast are found in the Mar Menor coastal lagoon and in the Ebro Delta (Figure 1) [2,10,11]. Unfortunately, both ecosystems are under considerable anthropogenic pressure and require urgent ecological restoration to recover their environmental status and ensure the survival of *P. nobilis*. The colonization of *P. nobilis* in the Mar Menor began in the early 1980s [12], and it quickly became a very abundant species (more than 1.5 million) [13]. However, since 2016, the lagoon has suffered severe episodes of eutrophic crises and environmental collapse, which have caused high mortality rates in fan mussels, among many other species [14,15]. *P. nobilis* in the lagoon have been threatened by various factors, such as the anchoring of boats, the impact of fishing nets, poaching, and some individuals even died after the entry and infection of *H. pinnae* due to a temporary decrease in salinity [13,16]. However, the sharp decline in the lagoon’s population was driven by eutrophication events [13,16]. The surviving population was estimated to be around 1500 individuals in 2021 [16].

For species with an advanced state of regression, both in situ and ex situ conservation strategies are proposed to reduce the threat against them. One of the common ex situ strategies is based on the maintenance and breeding of specimens in captivity to “save time” until the threat in the natural environment ceases, and also to establish a genetic reservoir to be used in active restoration programs when necessary [17]. The lack of effective ex situ reproduction represents a major bottleneck for the conservation and restoration of *P. nobilis*, as current captive breeding protocols remain limited in efficiency and reproductive success. Some studies on the cultivation of *P. nobilis* exist, although none have achieved juvenile settlement [18,19,20]. Trigos et al. [20] provided the first descriptions of the larval development of the species under captive conditions. Hernandis et al. [19] presented new insights into the maturation process of ex situ specimens, and Ferranti et al. [18] focused on transport and maintenance protocols, reporting spontaneous spawning and early larval development.

**Figure A1:**
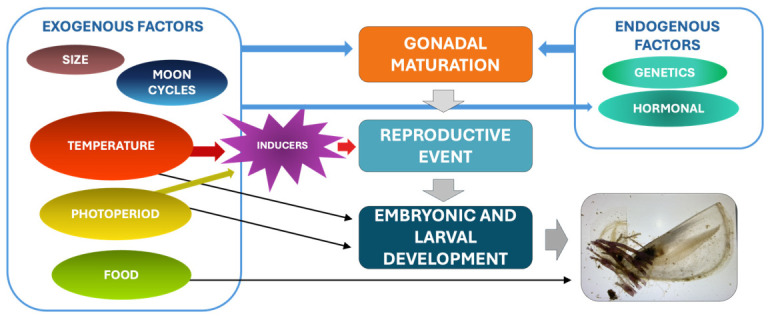
Exogenous and endogenous factors influencing the reproductive cycle of *Pinna nobilis*.

Endogenous and exogenous factors are involved in the maturation process of bivalves (Figure A1). Endogenous factors include genetic determinants, as well as the action of different hormones, which in turn depend on exogenous factors [21]. There are also different exogenous factors that influence the gametogenesis process, such as temperature, the abundance, quality and availability of food received, the photoperiod, lunar cycles, and even, according to some authors, the size of the individuals [22,23,24]. The quality and quantity of the food received by the broodstock is decisive in generating good quality gonads [25]. A deficient diet in bivalves can cause the weakening of the broodstock after the maturation process due to the mobilization of glycogen reserves stored in the muscle as an alternative to the usual reserves [26,27]. Furthermore, temperature is shown to be one of the main triggers of the conditioning or maturation process [28,29,30,31]. In *Atrina maura*, a species of the family Pinnidae Leach, 1819, temperature appears to be a determining factor in the activation of the reproductive cycle [32]. Likewise, maintaining an optimal temperature during the maturation process influences both the quality of the oocytes produced and the viability of the eggs after fertilization. In addition, the methodology of maturation in bivalve cultures is based on the exposure of the broodstock to changing temperature cycles during specific time periods [33]. The inducing effect of temperature on gametogenesis presents specific and geographic dependence, so that, depending on the species, the reproductive event can be activated in the laboratory with increases or decreases in temperature, or even generated by thermal shocks through applying maximum tolerance temperatures [19,20,23,25,26,30]. In contrast to the techniques that can generate stress in the reproductive specimens, this work proposes a strategy that begins with the observation of the maturation cycle of *P. nobilis* in the natural environment, specifically in the surviving specimens of the Mar Menor coastal lagoon, and then replicates it in the culture facilities as a naturalized process in order to achieve reproduction. This technique allows stress to be reduced in broodstock and even avoids the possible mortality of individuals subjected to more aggressive techniques, such as temperature shocks.

With the goal of ensuring the survival of *P. nobilis*, the main objective of this work is to advance the knowledge required to establish reproductive protocols for the species. This involves understanding its reproductive behavior in situ and transferring key parameters to achieve controlled maturation and reproductive events under ex situ conditions. Firstly, we proceeded to characterize the reproductive events in the Mar Menor lagoon. An exhaustive monitoring of the reproductive episodes of this species in the lagoon, and the variations in the environmental factors that characterize these episodes, was performed for three years. This in situ information provided the framework for determining the thresholds for exogenous factors in two controlled *P. nobilis* reproductive experiences, conducted in the laboratory in 2020 and 2023. Both experiences involved an ex situ emulation of the fan mussel’s reproductive cycle, including gonad maturation in ex situ and in situ conditions, and resulted in five different reproductive events.

## 2. Materials and Methods

### 2.1. Study Area and Collection of Adult Specimens

The Mar Menor is the largest hypersaline coastal lagoon of the Mediterranean basin, located in southeastern Spain (Figure 1) [34]. The long-term water temperature ranges from 10 to 32 °C and salinity can vary between 38 and 47 PSU [35], depending on weather conditions, although it usually remains stable at 45 PSU.

Between 2019 and 2023, in situ observations of the reproductive processes of *P. nobilis* in the Mar Menor were conducted, and significant progress was also made in developing captive breeding protocols for the species. The search of in situ gamete release events has focused on the surviving specimens in the lagoon after the 2016 environmental collapse [16], mainly in Barón Island, Pedrucho and Pueblo Cálido (Figure 1).

The specimens maintained ex situ for the different reproductive experiences were collected from Barón and Perdiguera Island and Pedrucho (Figure 1) under management permits INF/2020/0017 and AUF/2021/0008 issued by the General Directorate of Natural Resources of the Regional Government of Water, Agriculture, Farming, Fisheries, and the Environment. A total of 30 specimens of *P. nobilis* were randomly selected and transferred to the Murcia University Aquarium for ex situ maintenance, of which 21 were selected for the reproductive experiences and transferred to the maintenance and breeding systems. The nine specimens excluded from the breeding group were rescued from their original location after a disturbance was detected in the area where they were found. They were also showing signs of weakness. For this reason, they were kept at lower temperatures to avoid stimulating the activity of any potential pathogens. The transfer from the natural environment to the facilities was performed in accordance with the *P. nobilis* translocation protocol [36].

### 2.2. Identification of Reproductive Events and In Situ Gamete Collection in the Mar Menor Lagoon

The localization of the reproductive events in the natural environment was performed through systematic samplings focused on the best-preserved individuals of the lagoon [16]. Sampling days were intensified according to the temperatures observed as optimal for the release of gametes [37]. Once a reproductive event was detected for the first time, temperature and photoperiod were recorded and used as reference and threshold values for environmental parameters to adjust and design sampling campaigns in successive years.

The in situ collection of gametes for subsequent laboratory study was carried out by using 100 mL syringes, absorbing the emissions a few centimeters away from the spawner. The syringes were then transported in three hours to the laboratory at the University of Murcia Aquarium in coolers conditioned for transport in optimal conditions without temperature variations. Once in the laboratory, the eggs were observed and the percentage of fertilization was determined by homogenizing and counting the samples up to five times using video recordings. Fertilized eggs were identified by their movement, as they reach the ciliated gastrula or early trochophore stage after three hours [37].

### 2.3. Systems, Conditions and Ex Situ Maintenance of Broodstock

**Figure A2:**
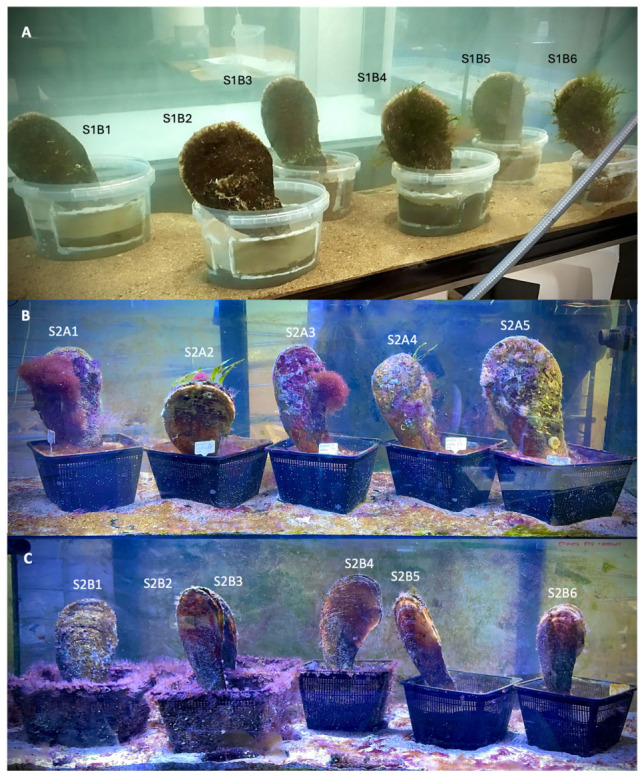
*P. nobilis* broodstock for 2020 experience (**A**). *P. nobilis* broodstock for 2023 experience (**B**,**C**).

Two broodstock maintenance recirculating water systems were designed (S1 and S2). Each system consisted of three maintenance tanks sharing a common filtration and temperature control system. The filtration system was designed as a sequence of different elements including mechanical, chemical, biological and germicidal filtration. Each tank had a volume of 980 L, and the total volume of the systems was 3440 L. Between five and six specimens were kept in each tank. The filtration flow rate in each tank ranged from 700 to 1500 L/h, depending on the time of day. In addition, two water recirculation pumps were installed in each broodstock tank to generate currents similar to those observed in the natural environment. The water used in the broodstock tanks was synthetic seawater, filtered at 1 µm and sterilized with UV germicidal lamps at sterilization level 5 [37]. Systems were designed to maintain groups of specimens in order to facilitate interaction during reproductive events. The specimens were placed inside the tanks in a vertical position, in baskets that allow oxygenation of the sediment (Figure A2).

**Figure A3:**
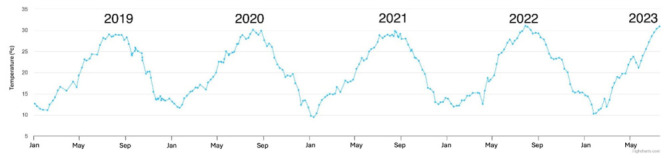
Evolution of mean temperature values observed in Mar Menor between 2019 and 2023. Source: Own elaboration based on https://canalmarmenor.carm.es (accessed on 15 October 2023).

**Figure A4:**
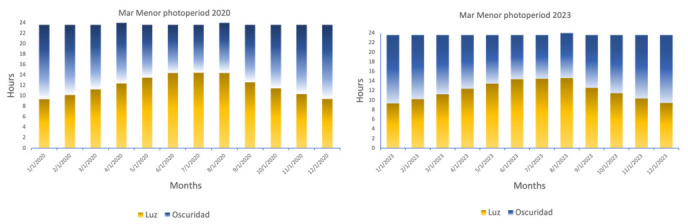
Mar Menor photoperiod in 2020 and 2023.

In both systems (S1 and S2), the conditions observed in the natural environment were reproduced for temperature and photoperiod (Figure A3 and Figure A4). The temperature cycle was regulated based on the thresholds observed in the Mar Menor (Figure A3), moderating extremes between 14 and 28 °C to prevent stressing the specimens [38], while ensuring that the conditions remained suitable for proper maturation. Reference data on the weekly temperature evolution in the Mar Menor were obtained from public data available on the web: https://canalmarmenor.carm.es/ (accessed on 15 October 2023). The annual variations in photoperiod were reproduced by programming the lighting equipment.

**Figure 2:**
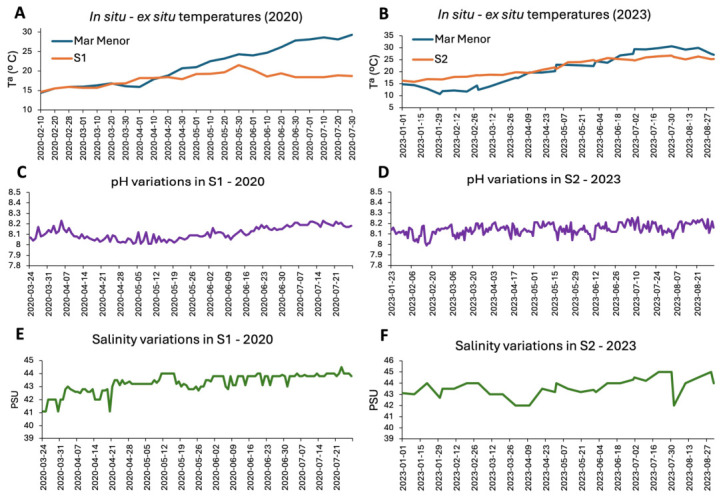
In situ and ex situ temperature variations during the 2020 and 2023 study periods (**A**,**B**). pH (**C**,**D**) and salinity (**E**,**F**) variations in ex situ laboratory systems S1 and S2.

Two reproduction trials were carried out: Experience 1 with S1 in 2020, and Experience 2 with S2 in 2023 (Table 1). Specimens in Experience 1 were subjected to mantle biopsies in an initial quarantine system [39] to detect possible infections. In contrast, this procedure was not performed in Experiment 2 because previous observations have shown post-biopsy mortality in some individuals [37]. Table 1 provides a summary of both experiences, including the number of individuals, their origin, size, environmental conditions maintained in the tanks, etc. Additionally, Figure 2 shows the variations in temperature, pH, and salinity in each of the systems. The experiments followed the gonadal maturation schedule described by Deudero et al. [27]. The maturation of female gonads was initiated in the natural environment and completed in the laboratory. However, in Experience 2, S2A broodstock were matured ex situ, in the Aquarium facilities, while S2B broodstock were matured in the natural environment to compare spawn quality and larval viability (Table 1).

Regarding feeding, the daily ration was divided into three doses, during which the water supply from the filtration system was cut off for two hours. The total feeding time was 6 h a day. In Experience 1, broodstock feeding throughout the reproductive cycle was based on a mixture of live phytoplankton and gels of the species *Tisochrysis lutea*, *Tetraselmis chuii* and *Phaeodactylum tricornutum*, at a rate of 40,000–70,000 cells mL^−1^, enriched with a base slurry made in the laboratory with various species of caridean crustaceans (*Palaemonetes varians*, *Palaemon elegans*), copepods (*Tigriopus* sp. and *Tisbe* sp.) and mysidaceans (*Mysis* sp.) administered at a rate of 240 mL per day via a continuous peristaltic pump. In Experience 2, the feeding was composed of three species of microalgae cultivated in the laboratory, two of which were fixed in the diet: *Tisochrysis lutea* and *Tretraselmis chuii*, while the other species alternated between the diatoms *Chaetoceros gracilis* and *Phaeodactilum tricornutum*. During Experience 1, the broodstock were fed a mixture of live phytoplankton and phytoplankton gel due to the gel’s ease of use compared to cultured phytoplankton and its success in the cultivation of other bivalve species; however, it was subsequently decided to use only live phytoplankton in Experience 2 due to the poor results obtained with the gel in the long-term maintenance of *P. nobilis* specimens. Phytoplankton strains came from the Marine Microalgae Culture Collection (CCMM) of the Institute of Marine Sciences of Andalucia (ICMAN–CSIC). The concentrations of each species varied according to their size: *Thisochrysis lutea*, *Tetraselmis chuii* and *Chaetoceros gracilis* or *Phaeodactylum tricornutum* were present in a ratio of 6:2:7. To dose the food, the same volume of culture was taken from each species. The 6:2:7 ratio refers to the number of cells per µl present in the selected volume. Once mixed and ready for dosing, there were 6 × 10^3^ *T. lutea* cells, 2 × 10^3^ *T. chuii* cells, and 7 × 10^3^ cells of the selected diatom (*C. calcitrans* or *P. cornutum*) for every 3 µL of culture. The daily dose in each tank of 980 L was calculated according to the approximation model of the diet proposed by Hernandis et al. [40] for *Pinna rudis*, with doses between 1.5 and 2.5 µm^3^ mL^−1^ (equivalent to 40,000–70,000 cell mL^−1^). The maximum was chosen in relation to the threshold of pseudofeces production [41]. The daily dose of phytoplankton was supplemented with two zooplankton species in order to provide the broodstock with an optimal nutritional profile rich in polyunsaturated fatty acids [42,43,44]. The species selected to feed the broodstock were a rotifer, *Brachionus rotundiformis*, selected due to its small size, and a copepod, *Acartia tonsa*, supplied in nauplius form. *B. rotundiformis* was fed in turn with the alga *Nannochloropsis gaditana*, whose nutritional profile is suitable for the requirements considered. Zooplankton was dispensed at a concentration of 4 to 5 units mL^−1^ and dosed at the same time as phytoplankton.

In both experiments, the state of gonadal maturation was assessed by observing the gonads through the open valves using dorsal visual observation. An increase in gonadal volume and even orange coloration of the gonads was observed in only 16% of the specimens with a valve opening greater than 2 cm. This method is harmless but imprecise, so intensive monitoring was necessary to accurately identify the timing of the reproductive event.

### 2.4. Induction, Gamete Release and Fertilization

After the maturation process, temperature variations observed in nature were emulated in the broodstock tanks until the threshold values identified as induction points were reached. The identification of the gamete release event was carried out by intensive and long-term monitoring of the broodstock. In order to detect the spawning events, egg collection filters with a 30 µm mesh were integrated into the filtration systems of the broodstock tanks. Likewise, and considering that gamete release occurs during the daylight hours (pers. obs.), visual monitoring was carried out from when the lights switched on at 8:30 a.m. until they went out in the breeding room at 9:00 p.m.

Gametes were collected directly from the broodstock holding tanks to avoid the stress associated with transferring specimens to other tanks [37]. In addition, keeping all specimens in the same tank also promoted synchronized spawning, enhancing gamete release from both sexes during group reproductive events. Gamete collection was performed by using a transparent tube of 10 mm internal diameter attached to a transparent hose of 10.5 mm internal diameter, through which the gametes expelled directly from the exhalant region were siphoned into a collection vessel. After collection, the sperm were filtered through a 15 µm mesh sieve and the eggs through an 85 µm mesh sieve, followed by retention through a 30 µm mesh sieve to remove possible contaminants. Filtration through sieves at all stages of larval development is important to keep the culture clean and healthy. In bivalves, size-based separation is typically achieved by filtering them through a series of sieves [23]. Once the eggs and sperm were obtained and filtered, these were tested for any deficiency [23]. Sampling was performed by homogenizing the mixture in the collection tank, taking four 1 mL samples and dropping each onto a methacrylate plate used for counting in the stereo microscope. A Leica microscope model DM750 and a Leica stereo microscope model S6D, both complemented with a Leica camera model MC170HD and a Leica stereo microscope model EZ4HD with built-in camera (Leica Microsystems, Wetzlar, Germany), were used for the study and review. Images and videos were obtained and processed using LAS (Leica Application Suite) EZ V. 3.4.0 (Video S1).

The external fertilization process began when the status of the gametes was verified. Spermatozoa were brought into contact with the eggs in the collection tank (Video S1). The sperm/egg ratio should be 10:1 to exceed 90% fertilization [21]; sometimes it can be a higher ratio, up to 15:1 and even 20–30:1, but it is not recommended to exceed it to avoid polyspermy [33,45]. After fertilization, the eggs were transferred directly to the different target tanks to continue with the larval development experiments, which will be addressed in future work. When only sperm release occurred, 250 mL samples were collected and stored at 4 °C, a temperature at which they can remain viable for up to three days according to Trigos et al. [20]. In this way, if only eggs were obtained in the next episode of gamete release, fertilization capacity could be available using the preserved sperm. The state of the sperm was continuously checked under a magnifying glass. The fertilization rate was calculated by analyzing samples of collected eggs (n = 3). Each sample was examined under Leica microscope (Leica Microsystems, Wetzlar, Germany) at 100× magnification 180 min after laying and a one-minute video was recorded using the microscope’s camera. If the eggs had been fertilized, one can observe different stages of embryonic development, which may vary from one breeding event to another. Typically, however, they would have reached the ciliated gastrula stage by this time. At this stage, it is easy to distinguish unfertilized eggs that show no development or movement from those that are moving or undergoing division.

## 3. Results

### 3.1. In Situ Breeding Event in the Mar Menor Lagoon

**Figure 3:**
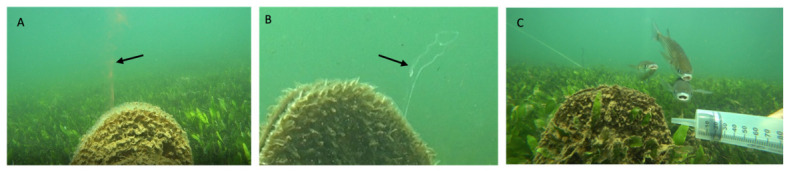
Specimen releasing oocytes (**A**), specimen releasing sperm (**B**) (black arrows), and golden gray mullets feeding on oocytes (**C**) 22/05/2019. (Photo: Javier Giménez.)

Three breeding events were recorded between 2019 and 2022, all located at Barón Island (Figure 1). The first observation took place on 22 May 2019 and was easily located by the presence of groups of golden gray mullets (*Chelon auratus*) attracted by the abundant number of released eggs (Figure 3C). The mullets were located on the upper margin of the fan mussel valves in the exhalant region, at the point of gamete release, feeding on them. The same behavior was not observed when male gametes were released. Although various parameters were monitored continuously, the study prioritized temperature and photoperiod as triggers of the reproductive event. The water temperature at the time of gamete release was 23.9 °C and the photoperiod 14:10 light:dark. Therefore, it was assumed that the temperature for the induction of the reproductive event was 24 °C, and this threshold was used as a reference for monitoring subsequent reproductive episodes in situ. On 29 May 2020, as well as on 19 May 2022, the release of male and female gametes by different specimens was detected again at similar temperatures of 24.4 °C and 24.3 °C, respectively (Figure 3A,B). During these spawning events, divers observed mullets (*C. auratus*) feeding on eggs near the exhalant siphon. However, the release of gametes was easier to detect on these occasions because the divers were on the lookout for it. The fertilization rate could only be calculated during the third spawning event because the first two samples were highly contaminated and had low viability once they were in the laboratory. Although both male and female gametes were collected, the female gametes had a fertilization rate of 90.2% ± 0.7% upon arrival at the laboratory; therefore, ex situ fertilization was not performed.

### 3.2. Specimen Maturation

Of the 10 specimens collected from the Mar Menor in 2020, 30% showed signs of mature female gonads upon arrival at the Aquarium facilities, based on a preliminary visual inspection. In individuals showing signs of possible gonadal maturation, egg release (grouped in mucous) was observed after mantle biopsy, likely triggered by the stress of the procedure. Specimens not initially identified as mature may have subsequently matured as males in the laboratory. In Experience 2, only 16% of the 11 individuals were identified as possibly maturing female gonads, while the remaining 80% showed no clear signs of female gonadal maturation.

**Figure 4:**
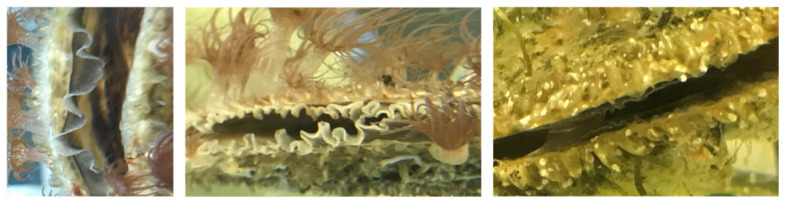
Different levels of white scalloped mantle edge in a mature specimen in the days prior to gamete emission.

Among the observations made during the experiences, external signs potentially associated with advanced maturation in *P. nobilis* were identified. Notably, changes in the typical arrangement and coloration of the mantle edge were observed, affecting 60% of the broodstock in Experience 1 and 87% in Experience 2 (Figure 4). This morphological change in the mantle has also been observed in wild specimens in the Mar Menor during May and June 2019, 2020, and 2022.

### 3.3. Induction, Gamete Release and Fertilization

In both experiences, 30% of the specimens released gametes of both sexes, always during daylight hours. All reproductive events begin with the release of male gametes by a single individual, which acts as the inducer of gamete emission in the rest of the group.

**Figure A5:**
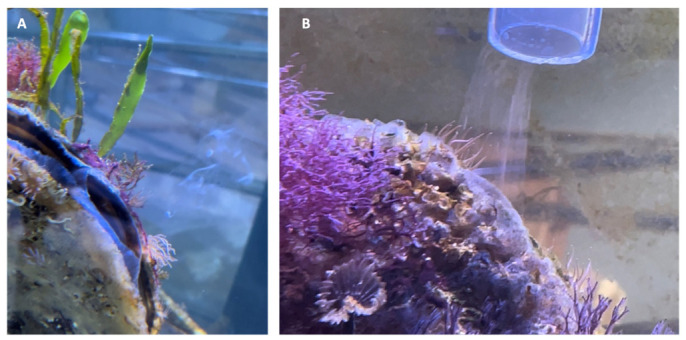
Fan mussel spawners at the time of gamete release. (**A**) Specimen releasing male gametes. In *Pinna nobilis* species, the release of spermatozoa follows a pattern of alternating pulses. (**B**) Specimen releasing female gametes. In *P. nobilis,* the release of oocytes is homogeneous from beginning to end. The photograph shows the egg collection tube for transfer to the fertilization buckets.

During Experience 1, the reproductive event started at a temperature of 21.5 °C and a salinity of 43.1 PSU. The gamete release process (Figure A5) took place over three consecutive days. Only one of the ten broodstock released eggs, while five released sperm (Table 2); the other four specimens showed no gamete release. On 10 June, sperm emission was detected from individuals S1B1 and S1B6. After 15 min, S1B4 began to release eggs. After a further 35 min, other specimens joined the reproductive event by releasing sperm—in this case, S1B5 from the same tank and S1A2 and S1A4 from tank S1A. In the results of the first egg sampling of specimen S1B4, an estimated 6350 × 10^3^ eggs were spawned, all of them homogeneously spherical with an average size of 60.00 ± 0.25 µm in diameter (Table 3). A minimum of 85% of fertilized eggs were detected; even so, the fertilization protocol was carried out and in the following sampling, a 98% fertilization was detected (Table 3).

In Experience 2, the temperature of the system at the beginning of gamete emission was 25.2 °C and the salinity was 41 PSU. Both values remained stable throughout the breeding event. During this experience, a total of four reproductive events were observed, each lasting between 2 and 4 days, and with a resting period between events of 3, 6 and 7 days. Of this entire group of spawners, 50% released only male gametes, no specimen released exclusively female gametes, while the remaining 50% released both male and female gametes, producing alternating gamete release with a minimum of 6 days between gametes of opposite sexes—as a result, they can be considered as simultaneous hermaphrodites with alternating gamete release (Table 2). Among all the specimens realizing both gametes, only S2B2 started releasing male gametes, while the rest of them started releasing female gametes (Table 2). Spawner S2B2 is remarkable, as it released sperm continuously for two full days during all daylight hours. In contrast, the rest of the broodstock exhibited sperm release limited to a maximum of four hours each morning. This specimen (S2B2) could be considered the “inducer” of the process: it started releasing sperm on June 14 at 10:17 and spent the whole day emitting pulses with high concentrations of male gametes. Once in darkness it stopped emitting gametes. On day 15, it continued with the release process from 9:16, coinciding with the end of the sunrise simulation programmed on the tank screen. This specimen continued releasing sperm at pulses similar to those of the previous day, until the sunset simulation programmed in the broodstock system and the rearing room was activated. The release of female gametes began on June 16. The case of the S2B1 breeder that started releasing eggs is also interesting: it released sperm after 6 days and 14 days later spawned eggs again, although this time a small amount was collected in its own pseudofaeces. A noteworthy case is that of S2A5, one of the specimens matured ex situ, which on June 21 produced slightly more than 3 × 10^6^ eggs, of which only 51 × 10^3^ could be collected, with a size of 50.33 ± 0.21 (Table 3).

In the sperm obtained and stored at 4 °C, viability and motility were observed up to the third day; however, in the sperm stored at room temperature (22 °C), a significant loss of motility was observed after 8 h.

## 4. Discussion

### 4.1. In Situ Reproductive Event in the Mar Menor Lagoon

Identifying the location of the in situ reproductive event required a significant sampling effort during the monitoring of the *P. nobilis* population in the Mar Menor. Specifically, the reproductive events were recorded in one of the areas with the highest fan mussel density in the lagoon [16].

The analysis of eggs collected in the natural environment showed that 90.2% were already fertilized. This suggests that the fertilization of the eggs, or a significant part of the process, took place in the pallial cavity, prior to their expulsion. Such fertilization would be favored by the filtration of water containing male gametes emitted by nearby specimens, which in turn had been activated by temperature, as suggested in Trigos et al. [20]. This fertilization mechanism could reinforce the theory of the gregarious patchy distribution of the species to ensure a successful reproductive cycle, where the probability of fertilization increases in grouped specimens [46]. It also suggests that isolated individuals are less likely to contribute to the genetic pool of the species.

When developing recovery strategies for *P. nobilis*, once the species’ reproduction protocol is established and viable seed has been obtained, areas occupied by isolated individuals should be considered for potential repopulation. These areas offer suitable conditions for adults, and the presence of reproductive individuals could enhance the genetic variability of the ex situ population once reintroduced into the natural environment. The presence of individuals in a specific area implies the possibility of the arrival of larvae from other areas (even if only occasionally), so the risk of genetic drift of the population resulting from captive breeding specimens decreases.

### 4.2. Maturation of Specimens In Ex Situ Experiences

The ex situ experiences described in this study included both complete and partial reproductive cycles, encompassing broodstock conditioning or gonadal maturation and gamete release, as well as instances involving only gamete release from mature broodstock collected from the natural environment. It may be interesting to elaborate either a complete reproduction protocol that covers from the maturation process to larval development, or a partial one, focusing the experience on gamete release and larval development, but with in situ specimen maturation. The partial protocol could be more efficient as individuals matured in the natural environment usually have more favorable exogenous and endogenous conditions than those provided in the laboratory [26]. Subsequent studies have also employed partial maturation with good results [18]. On the other hand, partial protocols are also more economical processes, because they do not involve developing and maintaining large-scale auxiliary cultures, which are necessary to condition the broodstock [33]. However, it is also necessary to advance knowledge and interpret certain results that arise from achieving the complete ex situ reproductive process.

As proposed in the work of Ángel-Dapa et al. [46] on the Pinnidae bivalve *Atrina maura*, the in situ viability of larvae depends on a conjunction of optimal factors throughout the reproductive cycle of the specimens. When all the environmental requirements do not converge in the reproductive events, it causes a certain percentage to have null larval viability. *P. nobilis*, belonging also to the Pinnidae family, seems to present certain parallelisms, which would explain why, in localities such as the Mar Menor, recruitment in *P. nobilis* seems to occur in pulses and populations are made up of homogeneous groups determined by age classes [37]. The settlement of juveniles takes place in moments when the ideal conditions converge in the different phases, which favour both a correct gonadal maturation stage and a period of optimal larval development [37]. A key point for the proper work of ex situ reproduction should be the identification of optimal thresholds of the main environmental factors that determine reproductive success in certain years and not in others [37,47].

Maturation in bivalve species is regulated by different endogenous and exogenous factors, of which temperature and photoperiod are particularly relevant [48]. According to Deudero et al. [27], based on work conducted in the Cabrera Archipelago Maritime-Terrestrial National Park (Western Mediterranean), the gonadal maturation chronogram of *P. nobilis* specimens in the Mediterranean follows different temporal patterns, with a time lag between females and males. Female gonads begin to differentiate in a small number of specimens between November and December, with most reaching an advanced stage of maturation between February and March, while males only begin to mature at that time. Nonetheless, the male gonads mature faster, and the reproductive period takes place between May and July [27]. This “lag” in the maturation of the gonads has also been observed in the present work. In Experience 1, the reproductive specimens were collected in February 2020, some of which already showed signs of maturation of the female gonad. A semi-complete breeding protocol was developed in Experience 1 that included the ex situ maturation of male gonads, while the female gonads only finished their maturation process in the laboratory. This type of conditioning is usually less demanding for male gonads than for female gonads. On the other hand, the conditions in the Mar Menor in February 2020 were not ideal at the time for the collection of specimens due to the processes of eutrophication and hypoxia [49], and it has not been possible to identify whether these poor in situ environmental conditions could have altered the gonadal maturation process of the specimens.

Several maturation methodologies were identified in the literature, mainly involving temperature-induced acceleration [19,23,50,51,52]. However, to avoid stressing the specimens, methods that could alter their natural life cycle were discarded. Instead, a naturalized maturation process was adopted, replicating the environmental conditions of their habitat.

In bivalve cultures, it is important to know the maturity stage of the broodstock before inducing gamete release [23]. In Experience 2, there were specimens that matured in the laboratory, together with specimens that matured in the wild. At the time of collection, the females showed signs of being mature, while the males were finishing their maturation process. There are different procedures to identify the maturity stage of broodstock. In commercial bivalve culture, one of the most common methods to observe the maturity stage is based on the study of the gonads of a representative group of specimens among the broodstock [23,26]. This procedure involves the death of specimens, so it is not extrapolable to experiences with *P. nobilis*. This study would have been enormously interesting and would have provided valuable data on the ex situ maturation process, comparable with existing data on the in situ maturation process of *P. nobilis* prior to the mass mortality [27], but ethically it would be highly questionable due to the current state of the population in the Mediterranean, and specifically the population in the Mar Menor. The method selected to work with *P. nobilis* must be wholly innocuous, so the evaluation of their state of conditioning by visual approximation was selected, consisting of the observation of the visceral mass from the upper region, taking advantage of the opening of the valves, in order to locate the gonad. This method is complex to perform and may not provide good results in a significant number of specimens. In the second experience, out of a total of eleven broodstock, gonadal development could only be assessed in two of them by visual dorsal approach. If it is not possible to determine whether the specimen is mature, it is advisable to proceed with thorough daily monitoring to detect the reproductive event.

Currently, one of the great challenges is to progress in the knowledge and optimization of the correct diets for adult and larval specimens, as this is one of the bottlenecks for the long-term ex situ maintenance of *P. nobilis* specimens [10]. Correct feeding is undoubtedly a determining factor. Changes and deviations from optimal conditions in the maturation process reduce the viability of larvae and the chances of reaching seed production [46,53]. In both experiences, individuals were provided with a diet based on phytoplankton and zooplankton, as has been done since 2018 with *P. nobilis* specimens maintained in the laboratory of the Murcia University Aquarium, and as suggested by the studies of [10]. The diets selected for *P. nobilis* broodstock, rich in polyunsaturated fatty acids, DHA (docosahexaenoic acid 22:6W3), and EPA (eicosapentaenoic acid 20:5w3 and arachidonic acid ARA), could prove essential in the formation of quality gonads [19,54,55] and likewise is particularly important for larval survival, especially during recruitment [56,57]. This fatty acid profile can be provided by certain species of phytoplankton, as well as by certain species of zooplankton. A study of the lipid profile of *P. nobilis*, as well as of laboratory-cultured phytoplankton and zooplankton species, is being prepared for publication. Zooplankton supplementation in the diet, based on species rich in polyunsaturated fatty acids, could become the key to long-term ex situ maintenance of fan mussel specimens; for this reason, it is important to continue with the work on maintenance protocols for the species (pers. obs.).

### 4.3. Induction, Gamete Release and Fertilization

The procedure for inducing spawning in bivalve cultures usually concerns aggressive methodologies for broodstock such as thermal shock, serotonin injection, or gonadal tearing [18,19,20,23,33]. None of these three methods can be considered for *P. nobilis*, since the first two result in a high mortality rate for adults and the third directly implies the sacrifice of the selected specimens. Therefore, a naturalized gamete release process was chosen based on natural inducers such as photoperiod and temperature, and later, the gamete release itself, which acts as another inductor element. Evidently, the sampling effort to locate gamete release is important at times when spawning is expected, but the result can be very different with induction procedures carried out with more aggressive methods due to the relationship between the optimization of natural inducing factors and the long-term viability of larvae [46,53].

In this naturalized system, the onset of gamete release, which in this study always begins with the emission by the inducer, stimulates the rest of the mature reproducers to release gametes, a behavior that is also observed in the natural environment. All episodes of gamete release were diurnal and spanned up to three consecutive days. The release of female gametes always occurred with a time lag of at least 15 min, enough time for sperm from adjacent specimens to enter the pallial cavity of the specimens with female gonads and be fertilized, activating the release of female gametes (most of them already fertilized). On the other hand, the discovery of new regulatory peptides involved in reproductive processes, such as maturation and spawning, in Pinnidae species like *Atrina pectinata*, offers new alternatives for inducing reproductive cycles in ex situ cultures ([58], Hashimoto K., pers. comm.).

Both in situ and ex situ observations showed that the release of male gametes tends to be pulsatile, with moments in which the density of spermatozoa is very high, followed by periods in which the density is very low, even null (Figure A5A). In contrast, in the case of female gametes, a constant release flow rate over time was observed, with a similar egg density throughout the expulsion process (Figure A5B). This contrasts with the way gametes are released in other bivalve species where sperm are expelled through the exhalant opening or siphon in a thin, steady stream, whereas the expulsion of eggs is more intermittent and are expelled in “clouds” [23].

In both experiences performed in this work, the importance of implementing an effective cleaning protocol for gametes, embryos and larvae was appreciated, with significant results in culture quality and larval viability ([23,59], Hashimoto K., pers. comm.).

After ex situ spawning, analysis of the samples showed a high fertilization rate, supporting evidence of internal fertilization [20], consistent with our observations of in situ fertilization. The sperm obtained maintains viability for up to three days, as suggested by Trigos et al. [20]. It is important that the sperm is stored at 4 °C, as storage at room temperature has shown to cause a significant loss of motility after 8 h.

Deudero et al. [27] identified *P. nobilis* as a species with successive hermaphroditism with asynchronous maturation. In later works such as by Trigos et al. [20], with individuals from the Embiez Archipelago it was observed how some specimens acted as simultaneous hermaphrodites, releasing male and female gametes simultaneously with self-fertilization processes. Prado et al. [60] and Hernandis et al. [19] identified the simultaneous maturation of both male and female gonads, and in their recent work they found individuals in the hermaphroditic phase with male and female gonads mature at the same time and with alternate and simultaneous release, both in the natural environment and in the laboratory. Simultaneous release of gametes is not effective because of problems in the larval stages related to self-fertilization [61]. In Experience 1 performed in this work, the release of male and female gametes was observed, acting as successive or sequential hermaphrodites with asynchronous maturation. However, in Experience 2, during the four reproductive events, simultaneous male and female gonadal maturation and the release of male and female gametes spaced in time were observed in four of the eight specimens. These specimens behaved as hermaphrodites with simultaneous maturation and alternate gamete release. The release of gametes in alternating mode is effective because it allows cross-fertilization to ensure larval viability, in addition to making the reproductive event more effective in a shorter period of time. Not a single specimen released both types of gamete simultaneously, thus eliminating the risk of self-fertilization.

Simultaneous hermaphroditism in bivalves has been associated with environmental stressors, particularly the presence of chemical pollutants [62,63]. In *P. nobilis* populations from the Ebro Delta, agrochemical contaminants such as pesticides and phytosanitary products may contribute to alterations in reproductive patterns [60]. Similar correlations have been documented in *Ruditapes decussatus* from the Mar Menor, where agricultural runoff near the *Albujón* wadi has been linked to physiological stress [64]. Further research is warranted to determine whether pollutant concentrations in fan mussel habitats are sufficient to induce stress-related reproductive changes, including hermaphroditism to its whole extent. Additionally, elevated temperature and salinity levels in the Mar Menor [65,66] may also act as stressors or selective pressures, potentially promoting hermaphroditism as an adaptive strategy. Comparable mechanisms have been observed in hermaphrodites such as *Tridacna* spp., where alternate gamete release avoids self-fertilization despite temporal proximity [67].

## 5. Conclusions

The *ex situ* reproduction of *Pinna nobilis* remains one of the main challenges for conserving this critically endangered species. Our findings demonstrate that successful reproductive conditioning in captivity depends heavily on recreating natural environmental cues. It may also benefit from partially maturing individuals in situ before transferring them to laboratory facilities. This study also sheds new light on the species’ reproductive strategy, supporting the existence of coordinated spawning mechanisms and suggesting adaptations that may enhance fertilization success while reducing self-fertilization. These observations improve our understanding of the reproductive biology of *P. nobilis* and provide valuable information for future conservation and restoration programs. Further research on spawning induction and larval viability is essential to optimize ex situ breeding protocols and support long-term population recovery efforts.

## Figures and Tables

**Table 1:** Summary of the two reproductive experiences (Exp). Ht (total length of *P. nobilis* individuals). Temperature (T^a^), pH, and salinity indicate the ranges of the parameters maintained in the tanks.

Exp.	Year	System/ Tank	N° Ind.	Collection Site	Ht (cm)	Maturation Start	T^a^ (°C)	pH	Salinity (PSU)	Feeding
1	2020	S1/S1A	4	Barón Island	37.4 ± 5.6	Mar Menor	14-0–22.0	8.02–8.24	41.0–44.5	Phytoplankton (mixture of live and gel) and zooplankton (base slurry)
1	2020	S1/S1B	6	Perdiguera Island	35.6 ± 2.5	Mar Menor				
2	2023	S2/S2A	5	Pedrucho	43.1 ± 2.8	Ex situ	15.6–27.7	7.99–8.26	42.0–44.0	Live phytoplankton and zooplankton
2	2023	S2/S2B	6	Barón Island	48.2 ± 2.7	Mar Menor				

**Table 2:** Reproductive events of Experiences 1 and 2. The inducer individual of each spawning event is indicated with an asterisk. The yellow and orange cells represent the release of spermatozoa and eggs, respectively. The green cells indicate the resting period between reproductive events.

**Experience 1** (in tanks S1A and S1B)									
Sperm	Eggs	*: Inducer, 21.5 °C, 43.1 PSU							
		**Breeding specimen**							
		**S1B1**	**S1B6**	**S1B4**	**S1B5**	**S1A2**	**S1A4**		
***Reproductive event 1***		Date 0: 10 June 2020			Time 0: 09:35				
Days	1	* 9:35	9:35	9:50	10:25	10:25	10:25		
	2								
	3								
**Experience 2** (in tanks S2A and S2B)									
Sperm	Eggs	*: Inducer, 25.2 °C, 41 PSU							
		**Breeding specimen**							
		**S2B2**	**S2B1**	**S2B5**	**S2B6**	**S2B3**	**S2A5**	**S2A4**	**S2B4**
***Reproductive event 2***		Date 0: 14 June 2023		Time 0: 10:17					
Days	1	* 10:17							
	2	9:16							
	3								
Rest period: 3 days									
***Reproductive event 3***		Date 0: 19 June 2023		Time 0: 09:35					
Days	1			*					
	2								
	3								
	4								
Rest period: 6 days									
***Reproductive event 4***		Date 0: 28 June 2023		Time 0: 09:30					
Days	1						*		
	2								
Rest period: 7 days									
***Reproductive event 5***		Date 0: 6 July 2023		Time 0: 09:19					
Days	1								*
	2								

**Table 3:** Data on the release of female gametes in both experiences (Exp.), (*n* = 5 for egg size and *n* = 3 for fertilization rate).

Exp.	Date	Specimen	Eggs Collected (N°)	Egg Size (µm)	Fertilization Rate
1	10 June 2020	S1B4	6.35 × 10^6^	60.00 ± 0.25	98.26% ± 0.62
2	16 June 2023	S2B1	10.84 × 10^6^	60.00 ± 0.07	91.00% ± 0.71
2	21 June 2023	S2A5	51.00 × 10^3^	50.33 ± 0.21	98.28% ± 0.66
2	21 June 2023	S2B3	24.39 × 10^6^	60.38 ± 0.25	98.18% ± 0.33
2	22 June 2023	S2B2	11.26 × 10^6^	60.02 ± 0.16	97.12% ± 0.35
2	6 July 2023	S2B1	1.16 × 10^6^	60.00 ± 0.22	45.08% ± 0.34

## Data Availability

The data presented in this study are available upon request from the corresponding author due to privacy and confidentiality restrictions.

## Supplementary Materials

The following supporting information can be downloaded at: https://www.mdpi.com/article/10.3390/biology15110847/s1, Video S1: Fertilized *Pinna nobilis* egg in contact with multiple spermatozoa.
